# Long noncoding RNA MALAT1 as a ceRNA drives mouse fibroblast activation via the miR-335-3p/P2ry2 axis

**DOI:** 10.1371/journal.pone.0308723

**Published:** 2024-08-12

**Authors:** Mengjie Chen, Jieying Peng, Guanghao Zhu, Cunhui Qian, Zhi Xiao, Xianmin Song, Haojun Yu, Rushi Huang, Wei Wang, Hongliang Zheng, Yafeng Yu

**Affiliations:** 1 Department of Otolaryngology Head & Neck Surgery, The First Afflilated Hospital of Soochow University, Suzhou, Jiangsu, China; 2 Department of Otolaryngology Head & Neck Surgery, Changhai Hospital of Navy Medical University, Shanghai, Shanghai, China; Hungarian Academy of Sciences, HUNGARY

## Abstract

Fibrosis is a complex pathological process that can lead to the permanent loss of biological function, with P2ry2 playing a crucial role in this process. Long non-coding RNAs (lncRNAs) have been reported to play an critically important role in the fibrotic process. However, it remains unclear whether lncRNAs can regulate fibrosis through P2ry2. In this study, we detected the expression of the long non-coding RNA metastasis-associated lung adenocarcinoma transcript 1 (lnc-MALAT1). We investigated the expression patterns of lnc-MALAT1 and P2ry2 in denervated skeletal muscle, a classical model of fibrosis. Additionally, we utilized a TGF-β-mediated fibrosis model in NIH/3T3 cells to examine the effects of lnc-MALAT1 and P2ry2 on fibroblast activation and the underlying regulatory mechanisms in vitro. Our results demonstrated that the expression levels of lnc-MALAT1 and P2ry2 were consistently elevated in denervated skeletal muscle, correlating with the degree of fibrosis. In vitro experiments confirmed the regulatory effect of lnc-MALAT1 on P2ry2. Furthermore, we identified miR-335-3p as a potential key molecule in the regulatory relationship of lnc-MALAT1/P2ry2. Dual luciferase reporter assays and AGO2-RIP verified the molecular sponging effect of lnc-MALAT1 on miR-335-3p. Additionally, we validated the regulation of the lnc-MALAT1/miR-335-3p/P2ry2 axis through experimental approaches. In conclusion, our study identified a crucial role of lnc-MALAT1/miR-335-3p/P2ry2 axis in fibroblast activation, providing a promising treatment option against the fibrosis.

## Introduction

Fibrosis is a process of bioremediation of tissue damage defined as the excessive accumulation of extracellular matrix (ECM) components such as collagen and fibronectin [1, 2]. It significantly impacts on tissue repair in all organs. When a tissue is injured, local tissue fibroblasts are activated and promote repair of the injured tissue through fibrosis. However, in cases of severe injury, excessive accumulation of fibrous connective tissue may result in permanent scarring, organ dysfunction and even failure. Permanent damage to organs caused by fibrosis is well known, such as the heart, kidneys, liver and skeletal muscle [3–6]. Therefore, identifying the underlying molecular mechanisms of fibrosis is key to the treatment of fibrotic tissues.

P2Y2 receptor (P2ry2) belongs to the purinergic G protein coupled receptors family [7], and has been shown to play important roles in the onset and progression of fibrosis in various organs, including the lungs, heart, and skin [8–10]. P2ry2 is the most highly expressed P2Y isoform in rat ventricular fibroblasts [11]. In addition, P2ry2 mediated most of the pro-fibrotic responses in rat cardiac fibroblasts under ATP stimulation [11, 12]. Our previous study demonstrated that ablation of P2ry2 alleviated skeletal muscle atrophy and fibrosis. Moreover, P2ry2 promotes skeletal muscle fibroblast proliferation and migration through AKT, ERK, and PKC [13]. However, the regulatory mechanism upstream of P2ry2 is still unclear.

Long non-coding RNAs (lncRNAs) are a diverse class of RNAs that engage in numerous biological processes [14]. As a competitive endogenous RNA (ceRNA), lncRNAs typically engage in post-transcriptional regulation by interacting with mRNAs or miRNAs in the cytoplasm [15]. Studies have shown that lncRNAs are involved in fibrosis in a variety of organs, including the liver, heart, lungs, and kidneys [16]. The metastasis-associated lung adenocarcinoma transcript 1 (MALAT1) is a highly conserved lncRNA that has been discovered as a prognostic marker for lung cancer metastasis [17, 18]. It has been reported to be expressed in almost all human tissues [19], with the highest expression in pancreas and lung, and abundant expression in skeletal muscle [20]. The lncRNA MALAT1 plays an important role in the fibrotic process of heart, liver, kidney and lung injury [21–25], However, its involvement in fibrosis is not yet fully understood, which could be a key point in identifying therapeutic.

In this study, we found that lncRNA MALAT1 markedly up-regulated in a mouse model of skeletal muscle denervation-induced fibrosis. We demonstrated that lnc-MALAT1 acts as a ceRNA for miR-335-3p, thereby regulating P2ry2 expression in vitro. Our study identified a critical role of the lnc-MALAT1-miR-335-3p-P2ry2 axis in fibroblast activation, provideing new insights for clinical understanding of the process and regulatory mechanisms of fibrosis.

## Materials and methods

### Fibrosis model in mice

A total of 25 C57BL/6 mice (male, 4-week-old and weighed 15–20 g) were purchased from Beijing Vital River Laboratory Animal Technology Co., Ltd, which were housed in an artificial 12 h:12 h light/dark cycle at 22–24°C in a relative humidity of 45–60%. They had free access to standard food and purified water. The 24 mice were divided into 4 groups, each containing 6 mice. Mice were anaesthetized via intraperitoneal injection of 1% sodium pentobarbital (0.5 mL/100 g) and immobilized on a plate. After that, the sciatic nerve of its right hindlimb was exposed and sheared. After 0, 1, 2 and 4 weeks, the mice were sacrificed by using CO_2_ asphyxiation chambers respectively, and the gastrocnemius of the operated side were used for follow-up RNA and protein examination and histopathological analysis. The gastrocnemius muscle removed immediately after surgery (0 weeks) was defined as the control group. All the animal experiments were approved by the Institutional Review Board of the Changhai Hospital (Shanghai,China). There were no clinical patients involved in this study, so no informed consent was obtained.

### Masson staining

After tissue collection, samples for histopathological analyses were fixed in 4% paraformaldehyde (Servicebio, Wuhan, China) and immersed in the fixative for 24 h. After embedding in paraffin, samples were sliced into 4-mm sections. Then, slices were applied for the hematoxylin and eosin (H&E) or Masson’s trichrome staining. Fibrotic area by Masson staining was evaluated using ImageJ. Three regions were analyzed on each slide.

### Cell lineage and cell culture

The mouse embryonic fibroblast cell line NIH/3T3 was purchased from Quicell Biology (Shanghai, China) and cultured in DMEM medium supplemented with 10% NBCS (YOBIBIO, Shanghai, China) and 1% penicillin and streptomycin. All cells were kept in 5% CO2 at 37°C.

### TGF-β-induced activation in mouse fibroblasts

TGF-β was purchased from Novoprotein, Suzhou, China. 10ng/ml TGF-β was added to NIH/3T3 cell line and mouse fibroblasts activation model was established after incubation for 24h.

### Cell transfection

The small interfering RNAs against lnc-MALAT1 (si-MALAT1), negative control (si-NC) and the targeting lnc-MALAT1 were all obtained from Shanghai GenePharma Co., Ltd., Shanghai, China. To overexpress or knockdown miR- 335-3p, micrON^™^ miR-335-3p mimics and micrOFF^™^ miR-335a-3p inhibitors were purchased from RiboBio (Guangzhou, Guangdong, China) and transfected into 3T3 cells. The transfection was carried out using the JetPrime Reagent (Polyplus Transfection, Illkirch, France) following the manufacturer’s protocol.

### Quantitative real-time PCR (qRT-PCR)

Total RNA was extracted from skeletal muscle tissue or cells using TRIzol reducing agent (Invitrogen) according to the instructions. RNA samples (1ug each) were reverse-transcribed by PrimeScript^™^ RT reverse transcription kit (Takara, Japan) to synthesize cDNA. On the LightCycler 480II real-time PCR system (Roche, Meylan, France), mRNA and miRNA expression were evaluated by qRT-PCR using the TB Green Premix Ex TaqII kit (TaKaRa, Japan) according to the manufacturer’s instructions. The expression of mRNA and miRNA was normalized using the expression of GAPDH and U6, respectively. All data were subjected to relative quantitative analysis using the 2-ΔΔCt method. The experiment was repeated 3 times.

### Western blot

RIPA lysis buffer was used to extract proteins from animal tissues or cells. Protein concentration was determined using Omni-Easy Instant BCA Protein Assay Kit (Epizyme, ZJ102). Proteins were separated on gradient gel (EpiZyme, LK309) and transferred to PVDF membranes, which were blocked for one hour with skimmed milk. After blocking, primary antibodies were used to detect P2ry2 (anti-P2ry2, Bioss, bs-4180R), α-SMA (abcam, ab7817), Collagen I (abcam, ab138492) and GAPDH (proteintech, 60004-1-lg). Subsequently, primary antibodies were conjugated by incubation with HRP-labeled secondary antibodies and colored by ECL STAR (Beyotime, P0018AM). Imaging was performed using a gel imaging system.

### Immunofluorescence (IF)

In brief, cells were washed 3 times with PBS and fixed with 4% paraformaldehyde. Triton X-100 (Beyotime, P0096) was used to permeabilize the cells.1.5% BSA was used to block the cells, after which the cells were incubated with α-SMA antibody overnight at 4°C. FITC-labeled secondary antibody was subsequently incubated. Cells were added DAPI stain to detect nuclei and results were visualized using fluorescence microscopy.

### Subcellular fractionation

Cytoplasmic and nuclear fractions were collected by NE-PER Nuclear and Cytoplasmic Extraction Reagents (Thermo Fisher, 78833). RNA was extracted from the respective fractionated fractions and subsequently detected using RT-qPCR to detect the expression levels of lnc-MALAT1, GAPDH, U6.

### Luciferase reporter assay

Wild-type (WT) and mutant (Mut) binding sites of miR-335-3p in the 3’-UTR end of the sequence of MALAT1 were subcloned into pmirGLO Dual-Luciferase vector to generate MALAT1-WT/Mut, which was subsequently co-transfected with miR-335-3p mimics into NIH-3T3 cells. Luciferase activity was assayed by the Dual-Luciferase Reporter Assay System.

### AGO2-RIP assay

The Imprint^®^ RNA Immunoprecipitation Kit was purchased from Sigma-Aldrich. Cells were extracted and lysed according to the instructions provided by the reagent vendor, after which anti-AGO2 antibodies (Sigma-Aldrich, MABE253) or IgG were added. The AGO2 antibody was subsequently recovered using magnetic beads. The expression of MALAT1 and miR-335-3p in the immunoprecipitates was subsequently detected by RT-qPCR.

### Statistics

Statistical analyses were conducted using GraphPad Prism 9 software. All biological replicates are expressed using mean ± SD. Comparisons between two groups were performed by using t-test or Kruskal-Wallis test, and comparisons between multiple groups were performed by using ANOVA or Mann-Whitney U test. P-values of less than 0.05 were considered to be significant.

## Results

### Skeletal muscle fibrosis while the expression of lnc-MALAT1 and P2ry2 upregulated after peripheral nerve injury

**Fig 1:**
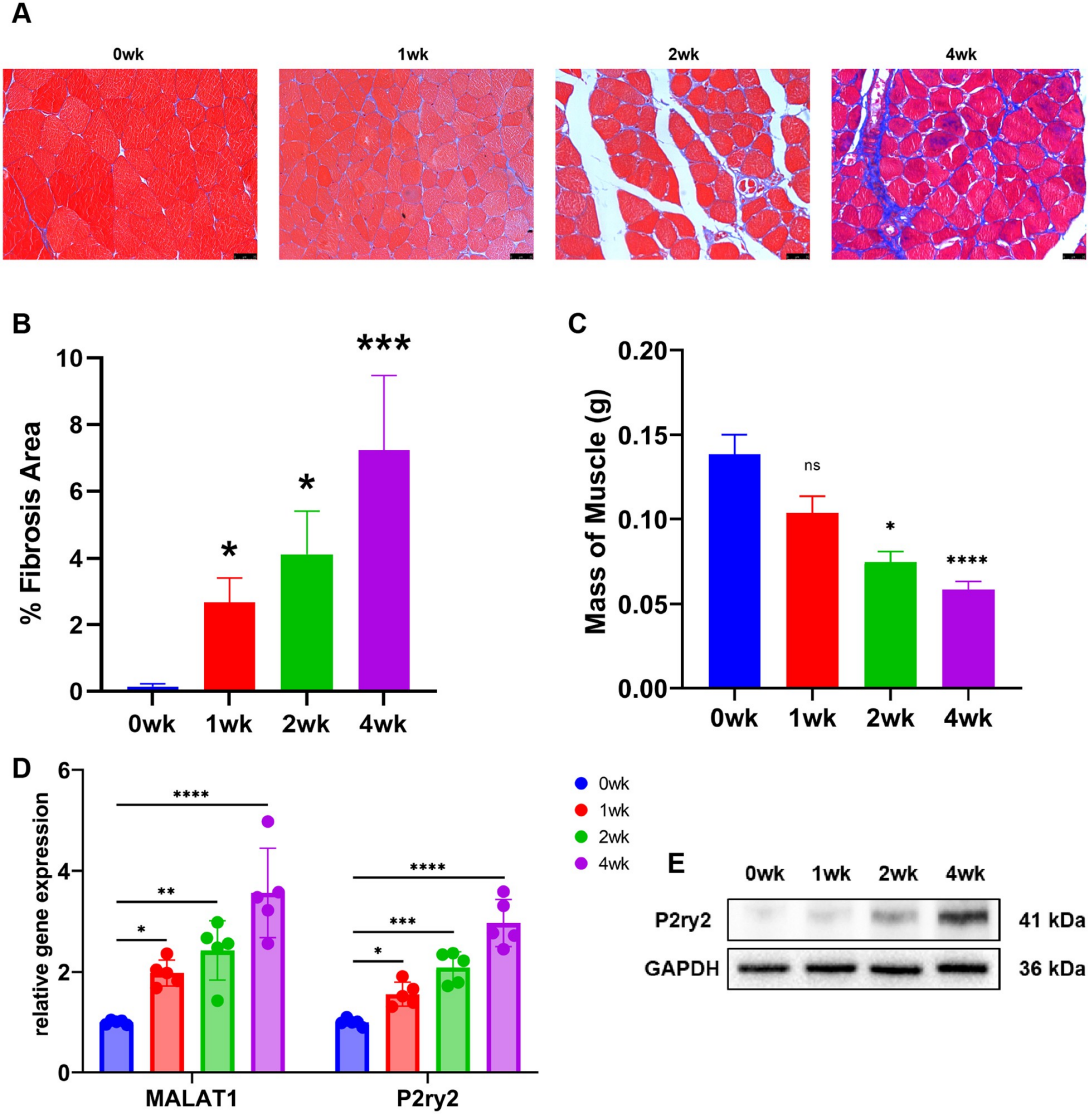
MALAT1 expression is upregulated in skeletal muscle after nerve injury. A, Image of Masson staining showing fibrosis areas of the gastrocnemius muscle in control (0 weeks) and 1, 2, and 4 weeks after nerve injury. B, Fibrosis areas of denervated gastrocnemius muscle measured by ImageJ. C, Relative muscle weight loss of denervated gastrocnemius muscle at different time points. D, mRNA expression of MALAT1 and P2ry2 in denervated gastrocnemius muscle measured by RT-qPCR. E, Protein expression of P2ry2 in denervated gastrocnemius muscle measured by Western Blot.

Consistent with our previous study, we constructed a model of peripheral nerve injury-induced skeletal muscle fibrosis in mice. Muscles were removed and weighed at 0, 1, 2 and 4 weeks after nerve injury. Following the severing of the sciatic nerve, masson staining of gastrocnemius showed that the area of collagen deposition in skeletal muscle increased consistently over time after nerve injury (Fig 1A and 1B). Correspondingly, the gastrocnemius showed a sustained loss of mass (Fig 1C). RT-qPCR was used to detect the expression of lnc-MALAT1 and P2ry2, while Western Blot was used to detect the protein expression level of P2ry2. Our results showed that the expression of lnc-MALAT1 and P2ry2 consistently increased following nerve injury (Fig 1D). Meanwhile, consistent with our previous findings, the protein expression level of P2ry2 consistently increased and was matched with the expression level of lnc-MALAT1 (Fig 1E).

### lnc-MALAT1 and P2ry2 were over expressed in TGF-β-treated mouse fibroblasts

**Fig 2:**
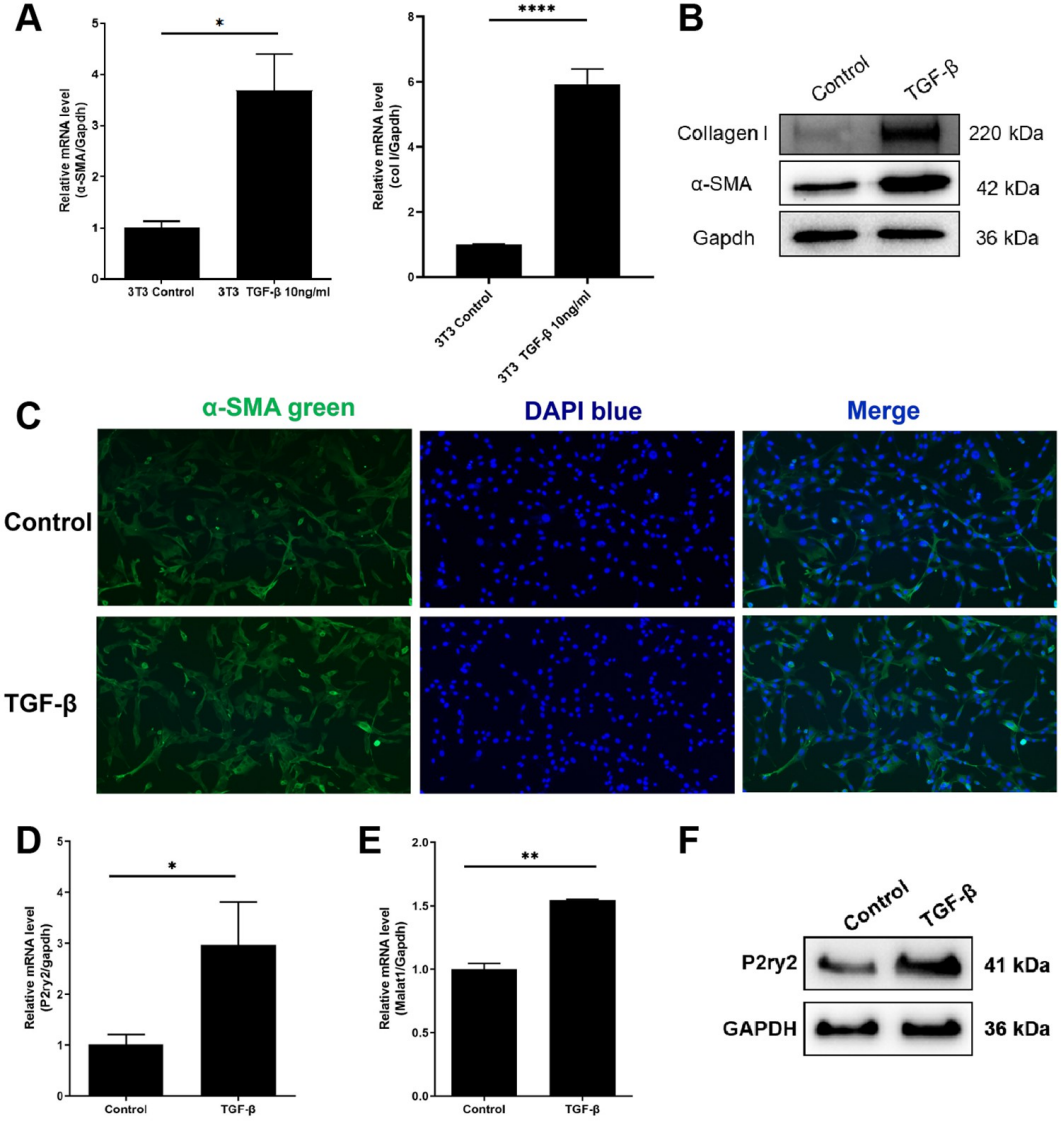
NIH-3T3 cells were activated to fibrosis by TGF-β while P2ry2 expression was upregulated. A, RT-qPCR showed that the mRNA expression of α-SMA in 3T3 cells was upregulated after adding 10 ng/mL of TGF-β for 24 hours. B, The expression of α-SMA and Collagen I was increased after adding TGF-β, detected by Western Blot. C, The expression of α-SMA was increased after adding TGF-β, detected by immunofluorescence. D, E, The mRNA expression level of MALAT1 and P2ry2 were upregulated after the cells were added with TGF-β, measured by RT-qPCR. F, The expression of P2ry2 was upregulated after the cells were added with TGF-β, measured by Western Blot.

We used TGF-β to induce NIH-3T3 activation in mouse fibroblasts. TGF-β stimulation induces the activation of fibroblasts in vitro, which has been well-established validated in previous studies [26, 27]. When 10 ng/ml of TGF-β was added, the mRNA expression of α-SMA in 3T3 cells was significantly increased (Fig 2A). The expression of α-SMA in 3T3 cells was also detected by Western Blot and cell immunofluorescence. Consistent with the mRNA level, the protein level of α-SMA and Collagen I in 3T3 cells increased significantly after being treated with 10 ng/ml of TGF-β for 24 hours (Fig 2B and 2C). The results, consistent with previous reports, represent the activation of the fibroblasts. The expression of lnc-MALAT1 was significantly increased after being added to TGF-β, consistent with the in vivo experiments (Fig 2D). The mRNA and protein levels of P2ry2 in 3T3 cells after TGF-β stimulation were consistent with changes in lnc-MALAT1 expression, as detected by RT-qPCR and Western Blot, respectively (Fig 2E and 2F).

### Reducing the expression of lnc-MALAT1 reduces the fibrosis of 3T3 cells

**Fig 3:**
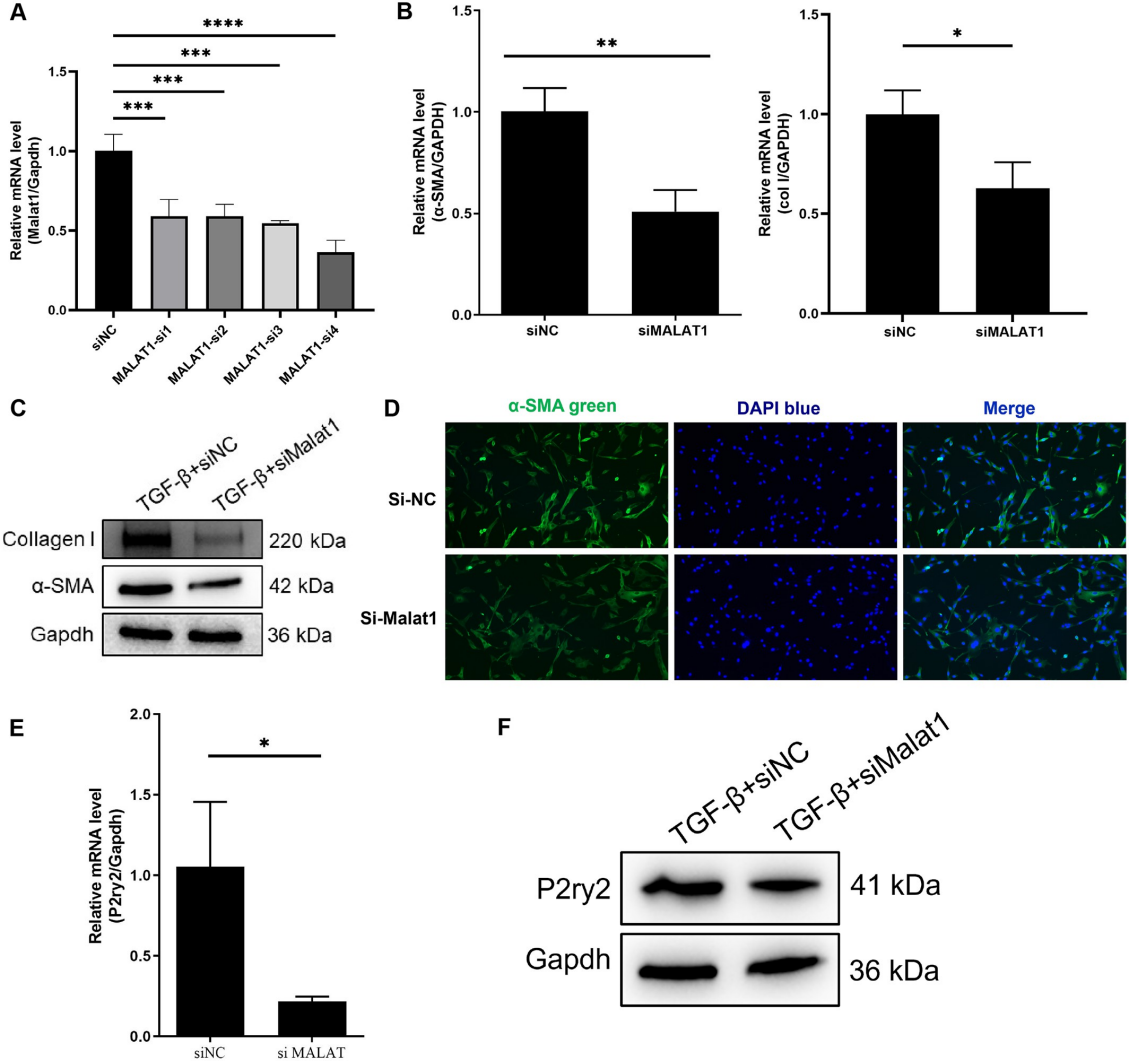
Reducing MALAT1 expression decreased fibrosis of NIH-3T3 and decreased the expression level of P2ry2. A, RT-qPCR showing the change of the expression level of MALAT1 in NIH-3T3 after transfection with siRNA targeting MALAT1. B, mRNA expression levels of α-SMA and Collagen I in NIH-3T3 fibroblasted cells transfected with siNC and siMALAT1, relatively. C, α-SMA and Collagen I expression levels in NIH-3T3 cells added with TGF-β and transfected with siNC and siMALAT1, measured by Western Blot, relatively. D, α-SMA and Collagen I expression levels in NIH-3T3 cells added with TGF-β and transfected with siNC and siMALAT1, measured by immunofluorescence, relatively. E, F, P2ry2 expression levels in NIH-3T3 cells added with TGF-β and transfected with siNC and siMALAT1, measured by RT-qPCR and Western Blot, relatively.

To investigate the role of lnc-MALAT1 in 3T3 cells, small interfering RNAs (siRNAs) were designed and used to interfere the expression of lnc-MALAT1. A total of 4 siRNAs were designed and were transfected into 3T3 cells respectively. RT-qPCR was used to detect the interference efficiency of siRNA. The results showed that MALAT1-si4 had the best interference efficiency, which could decrease the expression of lnc-MALAT1 to 36% at 24 hours (Fig 3A). MALAT1-si4 was named si-MALAT1 and was used for subsequent experiments. There was a significant decrease of α-SMA and Collagen I expression in cells transfected with si-MALAT1 compared with the negative control (si-NC) (Fig 3B). Meanwhile, Western Blot also showed that the protein expression levels of α-SMA and Collagen I were significantly decreased in the si-MALAT1 group (Fig 3C). After immunofluorescence of cells with added TGF-β, a decrease in the expression of α-SMA was found in the si-MALAT1 group compared with the si-NC group (Fig 3D).

To clarify whether MALAT1 has a regulatory effect on P2ry2, we used RT-qPCR and Western blot to detect the expression of P2ry2 in cells. The results showed that NIH-3T3 cells transfected with si-MALAT1 exhibited a significant decrease in P2ry2 mRNA levels compared to the si-NC group. Additionally, P2ry2 protein expression was also reduced (Fig 3E and 3F).

### MiR-335-3p may involved in the regulation of MALAT1-P2ry2 axis in fibroblast activation

**Fig 4:**
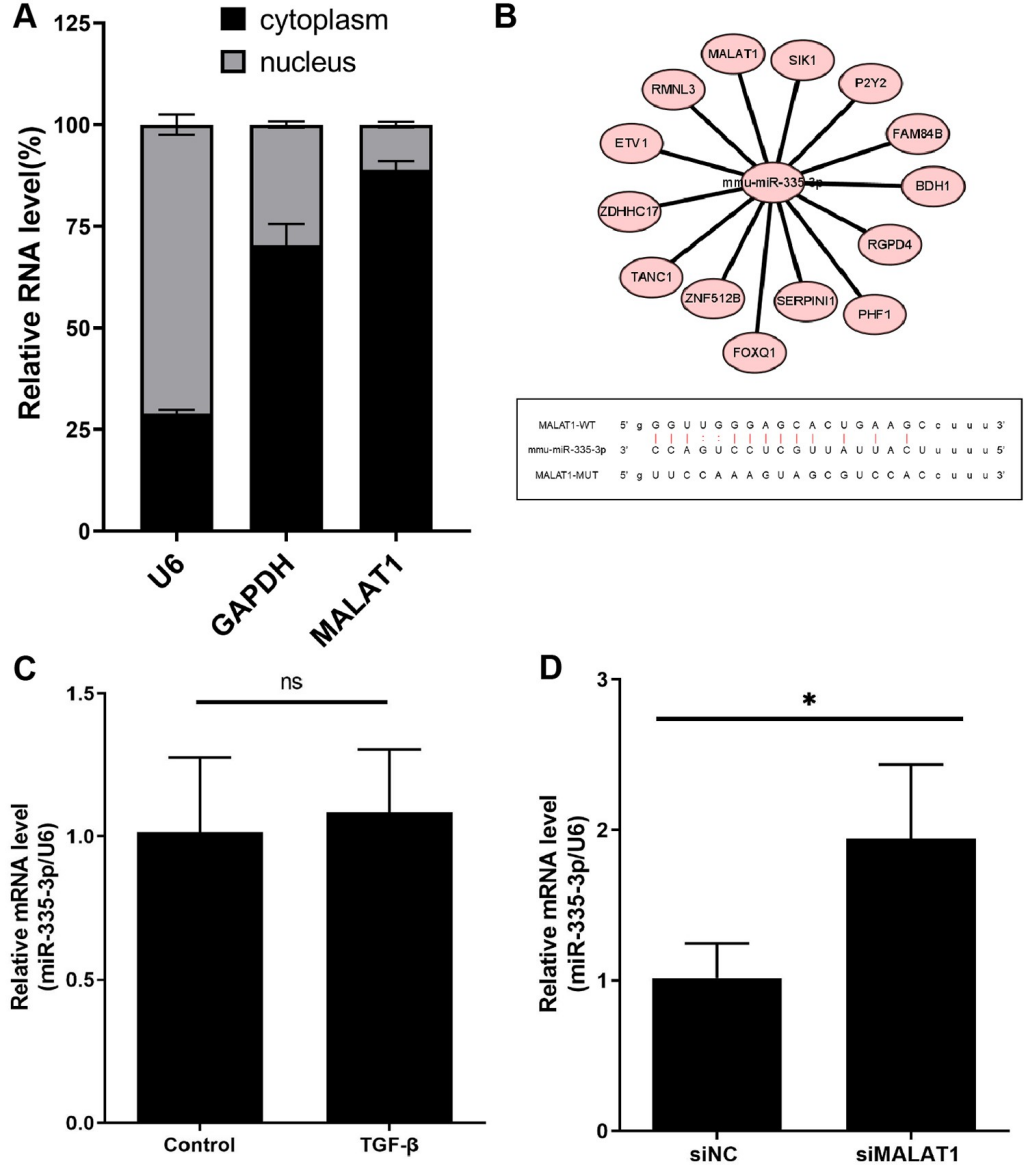
Prediction of a possible molecular mechanism of MALAT1 regulating P2ry2. A, Subcellular localization of MALAT1 in NIH-3T3 detected by RT-qPCR. B, Molecular interaction network and binding site of miR-335-3p prediction by RNAhybrid. C, Expression level of miR-335-3p in NIH-3T3 cells after adding TGF-β. D, Expression levels of miR-335-3p in NIH-3T3 cells after added TGF-β and transfected with siMALAT1.

To investigate the mechanism of lnc-MALAT1 function during fibrosis, we performed subcellular fractionation assays to determine the intracellular localization of lnc-MALAT1 in NIH-3T3 cells. The results showed that lnc-MALAT1 was primarily enriched in the cytoplasm of NIH-3T3 cells (Fig 4A). Based on this localization, we hypothesized that lnc-MALAT1 exerts its post-transcriptional regulatory function by acting as a molecular sponge for miRNAs. According to existing reports, miR-335-3p is potentially associated with the function of MALAT1 [28]. The binding targets of miR-335-3p were predicted by RNAhybrid 2.2 and Targetscan databases, and miR-335-3p was found to have possible binding sites with both lnc-MALAT1 and P2ry2 (Fig 4B). No significant difference was observed in the expression of miR-335-3p in 3T3 cells treated with added TGF-β (Fig 4C). However, after siRNA interfered with the expression of lnc-MALAT1, the expression of miR-335-3p showed a significant increase (Fig 4D).

### Lnc-MALAT1 as molecular sponge of miR-335-3p regulates P2ry2 in fibrotic 3T3 cells

**Fig 5:**
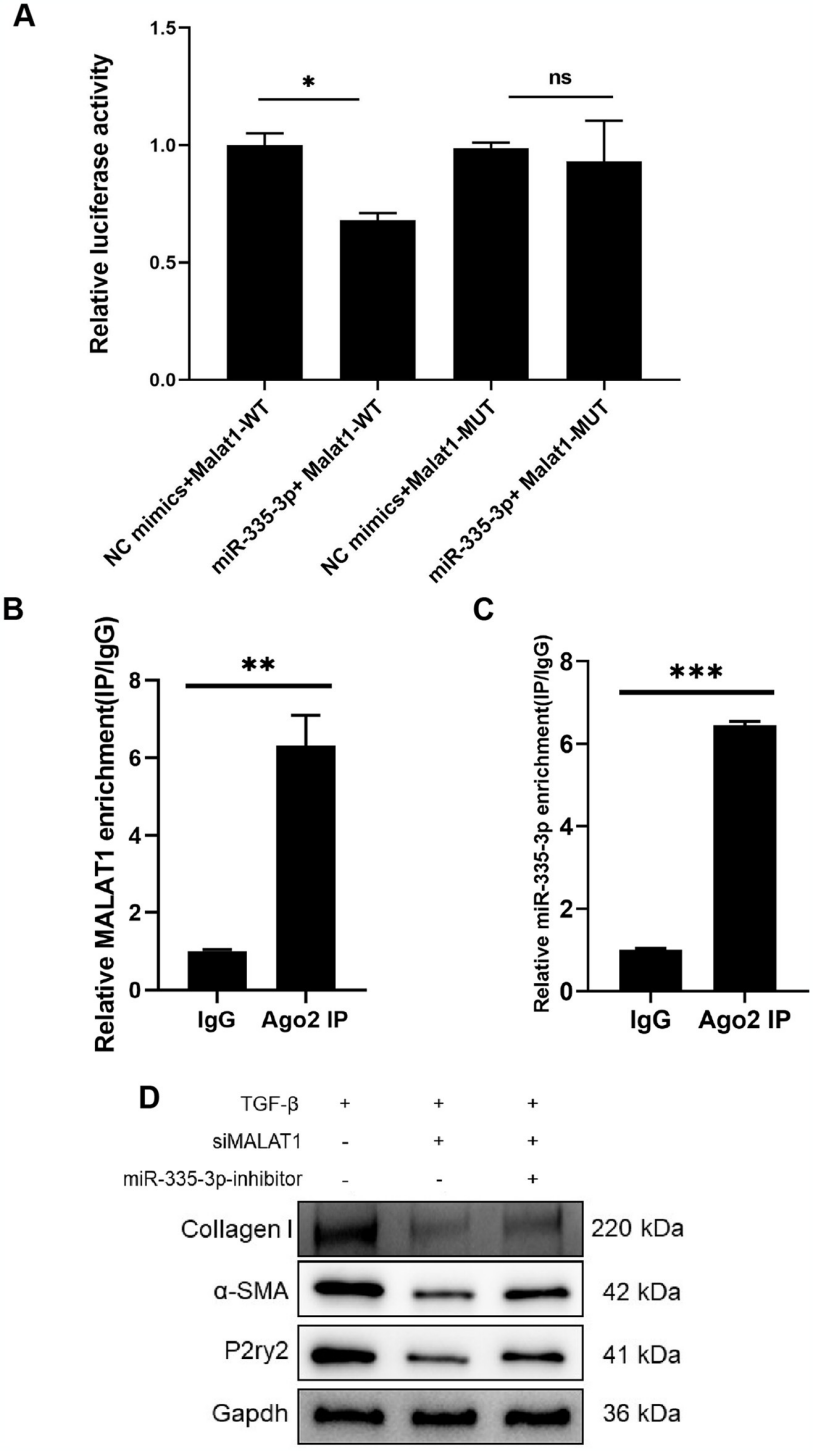
MALAT1 as ceRNA sponge binding miR-335-3p in NIH-3T3 cells. A, Dual-luciferase reporter assay was processed by co-transfection with miR-335-3p mimics and luciferase plasmids containing WT/MUT MALAT1 3′-UTR in NIH-3T3 cells. B, C, Ago2 immunoprecipitation was processed and the expression levels of MALAT1 and miR-335-3p in the precipitates were detected by RT-qPCR, relative to IgG. D, Western blot analysis showing the protein levels of α-SMA, Collagen I and P2ry2. Cells were treated with TGF-beta and transfected with si-MALAT1, miR-335-3p inhibitor or their negative control.

The wild-type or mutant binding cloning sites of lnc-MALAT1 were cloned into the PmirGLO vector and co-transfected with miR-335-3p and luciferase plasmid into cells. The luciferase reporter gene showed that up-regulation of miR-335-3p expression reduced the luciferase activity of PmirGLO-MALAT1-WT, but had no significant effect on the luciferase activity of PmirGLO-MALAT1-Mut (Fig 5A). More importantly, since the molecular sponge mechanism of miRNAs requires the involvement of Argonaute 2 (AGO2), we performed AGO2-RIP experiments and detected the expression of lnc-MALAT1 and miR-335-3p that were pulldown. The results showed that lnc-MALAT1 and miR-335-3p were significantly enriched in the AGO2 pulldown group compared with the IgG group (Fig 5B and 5C).

Meanwhile, we investigated the regulatory role of lnc-MALAT1/miR-335-3p on P2Y2 and fibrosis. As shown by Western Blot, protein P2Y2, α-SMA and Collagen I expression was down-regulated in the TGF-β+siMalat1 group compared to the TGF-β+siNC group, while P2Y2, α-SMA and Collagen I expression was restored in the TGF-β+siMalat1+miR-335-3p-inhibitor group (Fig 5D).

## Discussion

In this study we found that lnc-MALAT1 was significantly up-regulated in mouse skeletal muscle fibrosis, and experiment in vitro also demonstrated that lnc-MALAT1 expression was significantly up-regulated during fibroblast fibrosis. Silencing lnc-MALAT1 reduced TGFβ-induced fibroblast activation on 3T3 cells. Furthermore, bioinformatic prediction of miRNA targets, confirmed by dual-luciferase reporter and AGO2-RIP experiments, indicated that miR-335-3p could simultaneously target lnc-MALAT1 and P2ry2. Silencing of lnc-MALAT1 inhibits the expression of P2ry2. In conclusion, our study verified that lnc-MALAT1 could regulate the activation of fibroblast through the miR-335-3p-P2ry2 axis as a ceRNA to sponge miR-335-3p.

Preliminary findings from the current study revealed that lnc-MALAT1 is critical for maintaining tissue/organ fibrosis in vivo and in vitro. Several lines of evidence suggest that lncRNAs are aberrantly expressed in multiple tissue/organ fibrosis [16]. METTL3 boosts mitochondrial fission and induces cardiac fibrosis by enhancing LncRNA GAS5 methylation [29]. Lnc-TUG1 promotes pulmonary fibrosis progression via up-regulating CDC27 and activating PI3K/Akt/mTOR pathway [30]. LncRNA NEAT1/microRNA-129-5p/SOCS2 axis regulates liver fibrosis in alcoholic steatohepatitis [31]. LncRNA NEAT1 accelerates renal fibrosis progression via targeting miR-31 and modulating RhoA/ROCK signal pathway [32]. Lnc-MFAT1 promotes skeletal muscle fibrosis by modulating the miR-135a-5p-Tgfbr2/Smad4 axis [33]. These studies suggest that lncRNAs play an important role in the regulation of tissue/organ fibrosis. Consistent with previously reported lncRNAs, our study showed that lnc-MALAT1 is upregulated during tissue fibrosis. Additionally, lnc-MALAT1 silencing attenuated the expression of the fibrotic indicators and immunofluorescence positive area in vitro.

Moreover, the current study showed that lnc-MALAT1 could regulate fibrosis by acting as a miR-335-3p ceRNA. It was proposed that lncRNAs’ biological functions are closely related to their subcellular location [34]. Typically, lncRNAs localized in the cytoplasm regulate signaling pathways and mRNA stability or translation, while those localized in the nucleus are involved in RNA processing, transcriptional regulation, and chromatin interactions. The lncRNAs localized in the cytoplasm were suggested to regulate the target mRNA by acting as miRNA sponges. For example, Lnc-MFAT1 promotes fibrosis through sponging miR-135a-5p to regulate Tgfbr2/Smad4 axis in skeletal muscle [33]. Our results showed that the subcellular localization of lnc-MALAT1 was mainly enriched in the cytoplasm in 3T3 cells. The binding targets of lnc-MALAT1 and miR-335-3p were predicted by databases and confirmed by a luciferase reporter gene assay, consistent with the findings of Pouyanrad et al [28]. Our experiments demonstrate that lnc-MALAT1 functions as a post-transcriptional regulator by acting as a molecular sponge for miR-335-3p. In the present study, lnc-MALAT1 regulated the levels of fibrosis indicators through miR-335-3p, suggesting a regulatory role of the lnc-MALAT1-miR-335-3p axis in the activation of fibroblast.

Furthermore, P2ry2 was shown to be a target gene of miR-335-3p and plays an important role in mediating the fibrotic function of lnc-MALAT1/miR-335-3p. Previous data have shown that the P2Y2 receptor plays an important role in the development and progression of fibrosis in a variety of organs [8–10]. P2ry2 is the most highly expressed P2Y subtype in rat ventricular fibroblasts [11, 12]. Additionally, P2ry2 mediates the majority of pro-fibrotic responses of rat cardiac fibroblasts in response to ATP stimulation [12]. Stimulation of the P2ry2 can result in rapid and transient activation of MAPK/ERK [35]. Our previous articles have reported that P2ry2 promotes fibroblasts activation and skeletal muscle fibrosis through AKT, ERK, and PKC [11]. In this study, the pro-fibrotic effect of miR-335-3p inhibitor transfection was attenuated by P2ry2 silencing, suggesting that P2ry2 can mediate the antifibrotic effects of miR-335-3p. In addition, lnc-MALAT1 silencing inhibited P2ry2 expression, and inhibition of miR-335-3p restored its expression. This suggests an important regulatory role for the lnc-MALAT1/miR-335-3p/P2ry2 axis in the regulation of tissue/organ fibrosis.

However, there are limitations to this study. Our experiments demonstrated the role of lnc-MALAT1/miR-335-3p/P2ry2 axis in the regulation of fibroblast activation in vitro based on the mouse fibroblast cell line. However, cytology cannot fully represent pathological changes in disease. Studies in specific human diseases and animal models of fibrosis are still necessary. We are currently collecting fibrotic muscle specimens from patients in the clinic, and plan to validate the role of lnc-MALAT1/miR-335-3p/P2ry2 axis in the process of human muscle fibrosis in the future by RNA-seq as well as other experimental techniques. Additionally, our study has not been validated in an animal model, which is a critical step toward clinical translation. We plan to use adeno-associated virus to specifically knockdown or overexpress lnc-MALAT1 in mouse muscle in subsequent studies to observe its role in fibrosis and to provide a theoretical basis for future translational applications in the clinic.

In conclusion, our study revealed that lnc-MALAT1 was upregulated in fibrotic tissues. We demonstrated that lnc-MALAT1 can act as a miR-335-3p sponge to regulate P2ry2, leading to fibroblast activation through an in vitro cell line fibrosis model. This study provides new insights into the regulation of fibrosis by lncRNAs and suggests that lnc-MALAT1 is a promising therapeutic target.

## Supporting information

S1 Raw images (TIF)
