# Supplementary figures and images for: Long noncoding RNA MALAT1 as a ceRNA drives mouse fibroblast activation via the miR-335-3p/P2ry2 axis
Source: PLoS One. 2024 Aug 12;19(8):e0308723. doi: 10.1371/journal.pone.0308723 (PMC11318857; doi:10.1371/journal.pone.0308723)

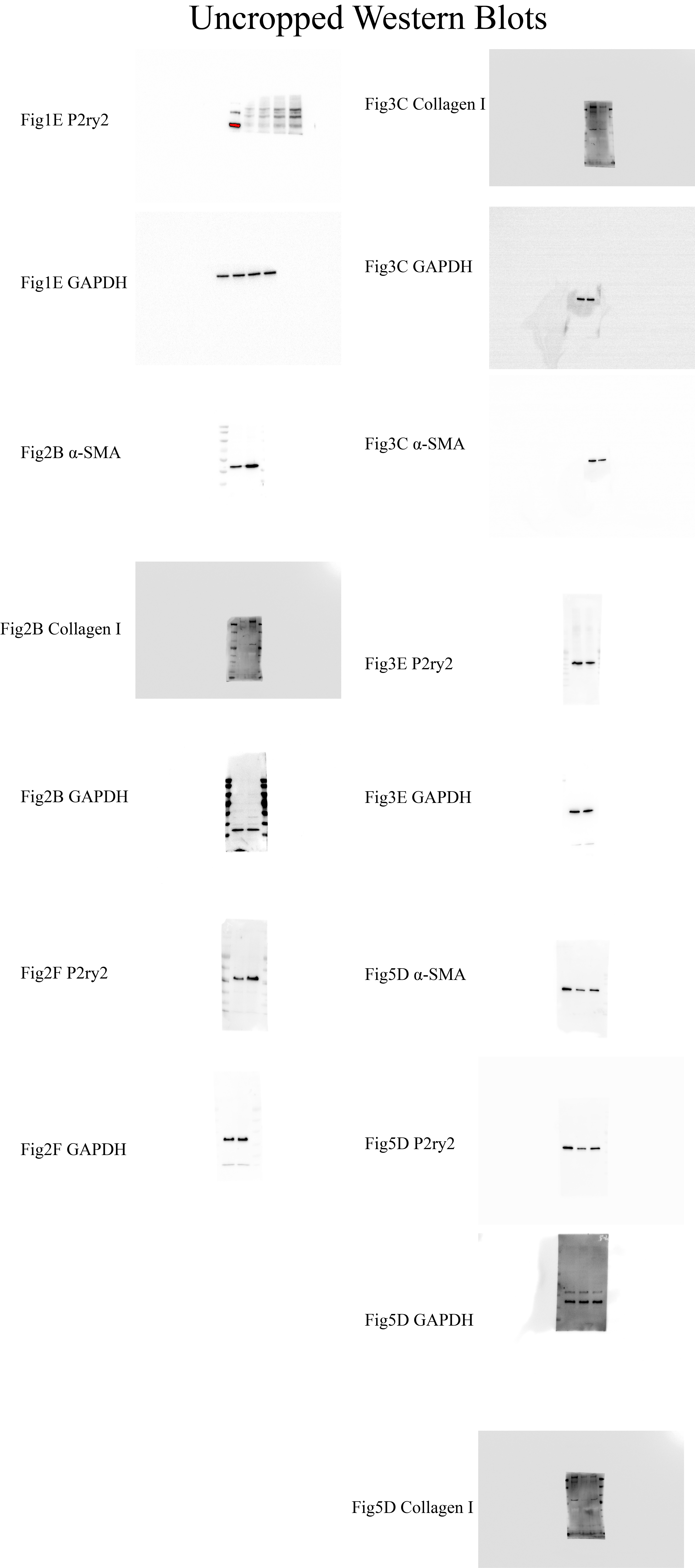

Supplement: S1 Raw images — (TIF) [file pone.0308723.s001.tif]
